# Transcriptome Analysis of Kiwifruit in Response to *Pseudomonas syringae* pv. *actinidiae* Infection

**DOI:** 10.3390/ijms19020373

**Published:** 2018-01-26

**Authors:** Tao Wang, Gang Wang, Zhan-Hui Jia, De-Lin Pan, Ji-Yu Zhang, Zhong-Ren Guo

**Affiliations:** Institute of Botany, Jiangsu Province and Chinese Academy of Sciences, Nanjing 210014, China; immmorer@163.com (T.W.); wg20092011@163.com (G.W.); 13915954315@163.com (Z.-H.J.); PPxsperfect@163.com (D.-L.P.); zhongrenguo@cnbg.net (Z.-R.G.)

**Keywords:** kiwifruit, bacterial canker, Psa, resistance

## Abstract

Kiwifruit bacterial canker caused by *Pseudomonas syringae* pv. *actinidiae* (Psa) has brought about a severe threat to the kiwifruit industry worldwide since its first outbreak in 2008. Studies on other pathovars of *P. syringae* are revealing the pathogenesis of these pathogens, but little about the mechanism of kiwifruit bacterial canker is known. In order to explore the species-specific interaction between Psa and kiwifruit, we analyzed the transcriptomic profile of kiwifruit infected by Psa. After 48 h, 8255 differentially expressed genes were identified, including those involved in metabolic process, secondary metabolites metabolism and plant response to stress. Genes related to biosynthesis of terpens were obviously regulated, indicating terpens may play roles in suppressing the growth of Psa. We identified 283 differentially expressed resistant genes, of which most U-box domain containing genes were obviously up regulated. Expression of genes involved in plant immunity was detected and some key genes showed differential expression. Our results suggest that Psa induced defense response of kiwifruit, including PAMP (pathogen/microbe-associated molecular patterns)-triggered immunity, effector-triggered immunity and hypersensitive response. Metabolic process was adjusted to adapt to these responses and production of secondary metabolites may be altered to suppress the growth of Psa.

## 1. Introduction

Kiwifruit bacterial canker disease was first reported on *Actinidiae chinesis* var. *deliciosa* in Shizuoka, Japan in 1984 [1]. In 2010, the pathogen *Pseudomonas syringae* pv. *actinidiae* (Psa) was detected in New Zealand, and within two years it infected 37% of New Zealand orchards and continues to increase [2]. To date, Psa has been detected in the main kiwifruit producing countries, including China, Chile, and European countries [3,4]. Pathovars of the species *P. syringae* cause important diseases in a wide range of plant species. To look for the way to control these diseases, researchers worldwide are trying to find the pathogenesis of *P. syringae*. Plants hold a complete immune system which is composed of two lines of defense. The first is PAMP-triggered immunity (PTI), which recognizes molecular microbial determinants, termed pathogen/microbe-associated molecular patterns (PAMPs/MAMPs), via pattern recognition receptors (PPRs) [5]. The second line is termed effector-triggered immunity (ETI) which detects injected effector proteins in the cytoplasm by resistance proteins and elicits further immunity. PTI and ETI can combine to cause hypersensitive response (HR) at infection site, which involves programmed cell death.

Studies on the pathogenesis of Psa are limited, but works on other pathovars of *P. syringae* especially the model species *P. syringae* pv. tomato DC3000 give us the chance to view the interactivity between *P. syringae* and the host. A functional hypersensitive response and pathogenicity (*hrp* pathogenicity island [PAI]) type III secretion system (T3SS) that directs the delivery of effector proteins into host cells has been shown to be the key pathogenicity factor required for *P. syringae* to colonize and parasitize host plants [6]. Plant immune system is a major target of type III effectors. *P. syringae* suppresses plant immune system by translocating immune-suppressing effector proteins through T3SS into plant cell [7]. Although effector proteins suppress immunity in some plants, in other plants, they trigger ETI [8] upon their recognition by cognate resistance proteins which, in turn, activate a secondary defense reaction HR [9].

The genome of Psa has been analyzed by different groups and genes possibly involved in pathogenesis were identified. McCann and his colleagues [10] identified 51 known type III effectors from four different clades of Psa and only 17 were found in all Psa genomes. This raised the possibility that the capacity to cause disease in kiwifruit resided primarily in the core genome of Psa. Meanwhile Psa also displayed a set of genes involved in degradation of lignin derivatives and other phenolics [11]. In-depth studies on Psa genomes have shown that this pathovar can rapidly adapt to a new host and new environments through the acquisition and/or loss of mobile genetic elements and virulence factors, thereby resulting in a multi-faceted plant pathogen [12]. In this study we analyzed the transcriptomic profile of kiwifruit infected by Psa, hoping to explore the response of kiwifruit on the molecular level and to lay foundation for understanding the pathogenesis of kiwifruit canker disease.

## 2. Results

### 2.1. De Novo RNA-Seq Assembly and Annotation of Unigenes

The valid reads from all samples were merged for de novo assembly using trinity software. A total of 110,134 unigenes with a N50 of 1226 bp were obtained (Table 1). All unigenes were longer than 200 bp and the average length was 759 bp. Functional annotation of unigenes were performed by blasting against various databases. Of all the 110,134 unigenes, 50,305 (45.68%) matched to known sequences, with 49,897 (45.31%) matching to sequences in Nr (non-redundant protein sequences) database, 34,331 (31.17%) matching to Swissprot database, 30,430 (27.63%) matching KOG (Clusters of eukaryotic ortholog groups of proteins) database, 20,524 (18.64%) matching KEGG (Kyoto encyclopedia of genes and genomes) database.

### 2.2. Functional Classification of Unigenes

**Figure 1:**
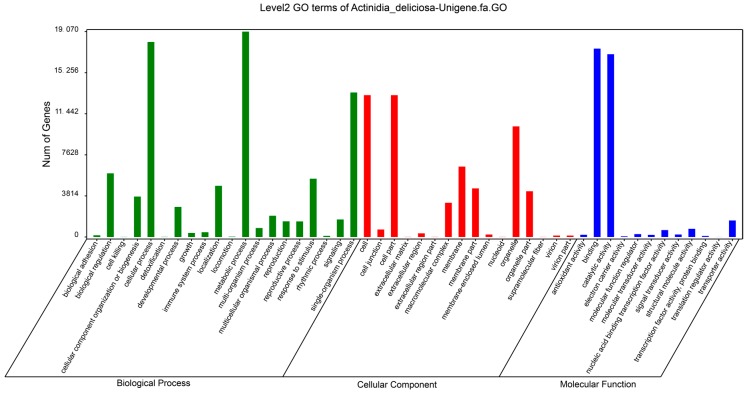
Gene Ontology classification of unigenes.

To better understand functions of the unigenes, we did GO (Gene Ontology) analysis and categorized the 20,524 unigenes matching to KOG database into three GO trees (biological processes, cellular components, and molecular functions), which were further classified into 48 functional groups (Figure 1). The three groups with the most number of unigenes in the category of biological process were cellular process, metabolic process and single-organism process. Groups with the most unigenes in cellular component were cell, cell part and organelle. Binding and catalytic activity were the biggest groups in molecular function.

### 2.3. Analysis of Differentially Expressed Unigenes

**Figure 2:**
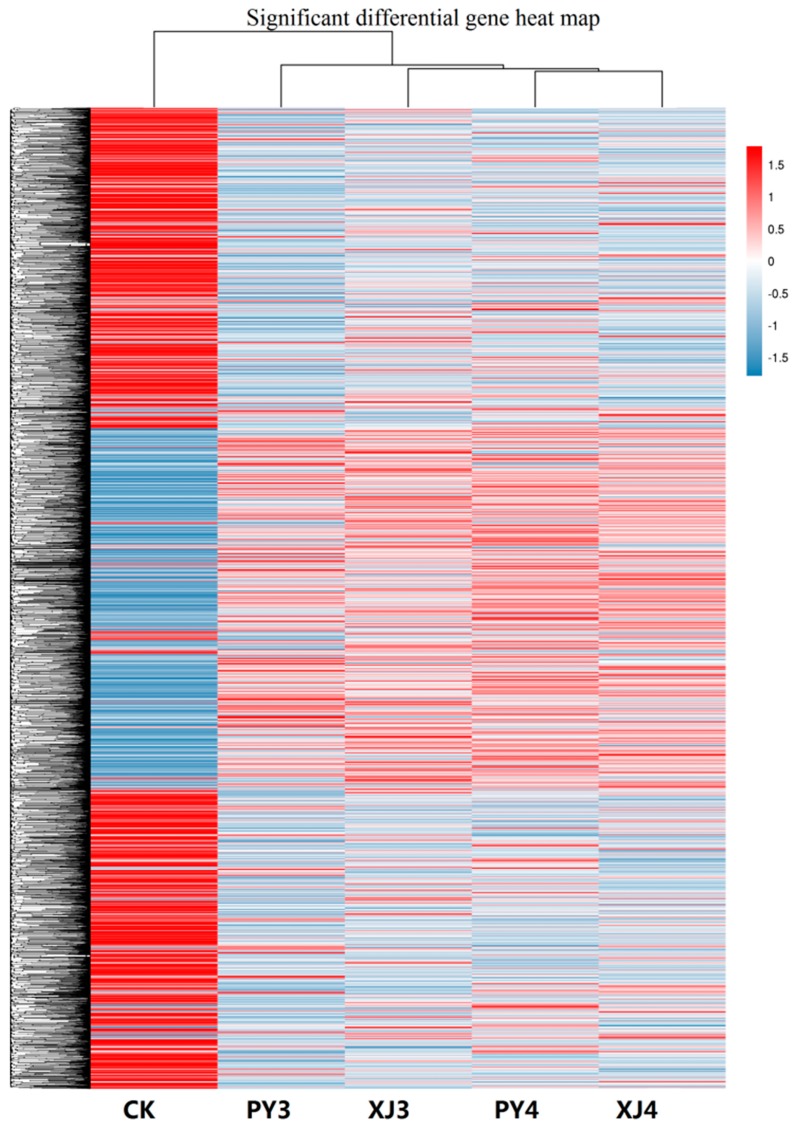
Cluster of significant differentially expressed genes of the five experimental samples. The RPKM (reads per kb per million reads) values of unigenes were used for hierarchical cluster analysis. Expression level was showed by different colors, the redder the higher expression and the bluer the lower. Five treatments were set: CK, only carved; PY3, inoculated with water and sampled 48 h after inoculation; PY4, inoculated with water and sampled at 96 h; XJ3, inoculated with Psa and sampled at 48 h; XJ4, inoculated with Psa and sampled at 96 h.

Expression level of each unigene was calculated and differentially expressed genes (DEGs) between Psa-treated sample and control at 48 and 96 h time points were identified. Cluster analysis of unigenes of the five samples was done (Figure 2). Compared with the other four samples, expression pattern of CK was obviously different and presented the most number of DEGs. PY3, XJ3, PY4, and XJ4 gradually showed more expression differences.

Twelve unigenes were randomly selected for qRT-PCR analysis (Figure S1) to validate the results of the RNA sequencing data. All of the selected unigenes exhibited similar expression patterns to those from RNA sequencing data, so indicating that the results of RNA sequencing were credible.

### 2.4. Functional Classification of Differentially Expressed Genes

**Figure 3:**
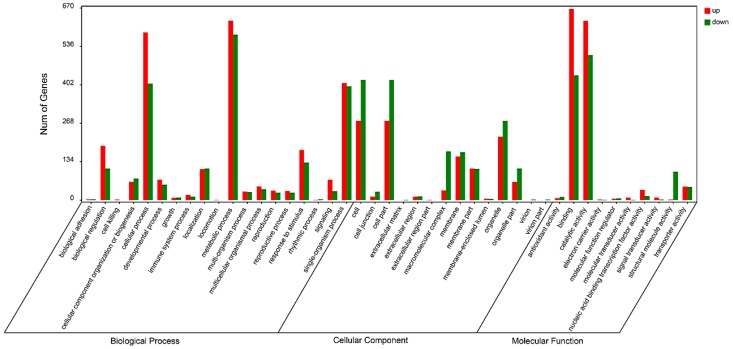
GO classification of differentially expressed genes.

Enrichment and classification of the DEGs were performed by searching GO and KEGG database (Tables S1 and S2). In the category of biological process, GO terms of cellular process, metabolic process and single-organism process enriched the most DEGs (Figure 3). In the category of cellular component, cell, cell part and organelle part enriched the most DEGs. Binding and catalytic activity got the most DEGs in the category of molecular function. Most of GO terms in the category of biological process had more DEGs up regulated by Psa treatment than those down regulated, and it was the opposite case in the cellular component category.

We compared the expression patterns between Psa-treated samples and control at 48 and 96 h respectively. There were totally 8255 (7.50%) DEGs (Table S3) between PY3 and XJ3, of which 2733 DEGs were down regulated in XJ3 relative to PY3, and 5522 DEGs were up regulated. Only 4281 DEGs (Table S4) were identified between PY4 and XJ4, and the numbers of DEGs down regulated and up regulated were similar. Among the most differentially expressed genes between PY3 and XJ3, we identified DEGs participating in terpene synthesis, salicylic acid-binding, jasmonate, disease resistance, ethylene response and WRKY transcription factor which were all related to disease resistance of plant. There were also many DEGs participating in biosynthesis of secondary metabolites, environmental adaptation and carbohydrate metabolism.

The top 20 progresses influenced by Psa treatment were summarized in Table 2. The top one and top four progresses were both related to terpene metabolism. Metabolic process enriched the most abundant DEGs (1203) in the 20 progresses. Four of the 20 progresses: regulation of defense response, regulation of response to stress, response to bacterium, and regulation of multi-organism process were directly related to plant response to stress. KEGG analysis resulted that the pathway of translation was the mostly influenced pathway and enriched the most abundant DEGs in the top 20 pathways. Other pathways influenced by Psa treatment included metabolism of terpenoids, polyketides, and other secondary metabolites, and metabolism of carbohydrate, amino acid, and lipid.

Multiple pathways involved in the metabolism of secondary metabolites were enriched, including those related to metabolism of the three main kinds of secondary metabolites: terpenes, phenols, and alkaloids (Table 3). Terpenes are the richest natural production and all of the monoterpenoid, diterpenoid, triterpenoid, and sesquiterpenoid (carotenoid) and terpenoid backbone biosynthesis pathways were regulated by Psa infection. Expression of as many as 64 (6.92%) genes in phenylpropanoid biosynthesis pathway which is the key progress in biosynthesis of phenols was regulated. Gene encoding the key enzyme phenylalanine ammonia-lyase (PAL) in phenol biosynthesis was found to be up regulated. Meanwhile, metabolism of the three main intermediate products tryptophan, tyrosine, and phenylalanine of phenol biosynthesis was also influenced. Two pathways in alkaloids biosynthesis were changed but the number of genes regulated only accounted a small part of all the genes annotated.

Pathogenesis-related (PR) proteins are thought to participate in plant-defense mechanism. We searched the kiwifruit transcriptomic profile and identified genes encoding PR proteins belonging to 10 families of the all 17 PR protein families (Table 4 and Table S5). We identified the most unigenes in PR-9, PR-5 and PR-14 family with properties of peroxidase, thaumatin-like and lipid-transfer protein respectively. Unigenes belonging to PR-1, PR-4, PR-5, PR-9, PR-10, and PR-12 showed differentially expression in Psa-infected kiwifruit. Meanwhile we also identified genes encoding three kinds of antimicrobial peptides: hevein-like peptide, knottin-type peptide and snakin peptide, of which hevein-like peptide and snakin peptide were regulated by Psa infection.

We screened out the resistant genes from the RNA sequencing data by searching the R-Gene database (PRGdb). In total, 4773 resistant genes were identified which could be grouped into 22 classes. The biggest class was RLP which acts as receptors to recognize avirulence genes. Of all the resistant genes identified, expression of 283 was changed by Psa treatment (Table 5). The class with the most differentially expressed resistant genes was NL which holds domains NBS (nucleotide-binding site) and LRR (leucine-rich repeat). Classes CN, CNL, N, and TNL also hold the NBS domain. Classes CNL, NL and TNL hold the LRR domain while RLK, RLK-GNK2, and RLP hold an extracellular leucine-rich repeat (eLRR). So, most of the differentially expressed resistant genes hold a NBS domain or a LRR domain. For most of the big classes, a certain number of genes exhibited regulated expression patterns in XJ3. Of the resistant genes, 211 (Table S6) hold the U-box domain and 25 were differentially expressed in Psa infected kiwifruit. Of the 25 differentially expressed U-box domain containing genes, 22 were up regulated in Psa infected kiwifruit and only three down regulated with small extent.

### 2.5. Analysis of Genes in Plant-Pathogen Interaction

From KEGG analysis, we enriched genes participating in the pathway of plant-pathogen interaction. A total of 593 related genes were identified (Table S2) and they involve almost all of the processes of the plant-pathogen interaction pathway, including PTI, HR, stomatal closure, ETI, and programmed cell death. Of all the 593 plant-pathogen interaction related genes, 59 showed altered expression in Psa treatment (Figure S2). Two DEGs encoding a pattern recognition receptor (PRR) *CERK1* were identified, but it belonged to PRRs which recognize chitin PAMPs of fungus. Expression of a protein kinase encoding gene *CDPK* was also regulated. It plays important roles in regulating gene transcriptional changes and other cellular response. Two transcription factors *WRKY25* and *WRKY29* also displayed changed expression. They participate in the MAPK (mitogen-activated protein kinase) signal pathway and induce expression of defense-related genes. The other differentially expressed transcription factors were pathogenesis-related genes transcriptional activator *Pti1* and *Pti4*. The *PR1* gene which plays an important role in plant disease resistance takes part in multiple biological processes including MAPK signaling pathway, plant hormone signal transduction and plant-pathogen interaction. Its expression change in Psa treatment indicated its disease resistance function. We also identified two differentially expressed disease resistance genes *RPM1* and *RPS2*. They both function with another resistance gene *RIN4* and induce hypersensitive response. Expression regulation of the above resistance genes in the plant-pathogen interaction pathway indicates that Psa treatment induced plant immunity and led to functioning of the related resistance genes to protect the plant body.

## 3. Discussion

Plants are organisms which cannot move like animals and therefore they cannot escape potential threats from environment, including pathogens, arthropods, and abiotic stress. They survive depending on constitutive physical and chemical defense mechanisms such as waxy cuticles, cell walls, and phytoanticipins [13]. Besides these common defense mechanisms, plants also evolve a specific immune system, namely PTI and ETI to defend themselves against various pathogens around them [14]. To successfully infect a plant, pathogens have to penetrate the physical layer and make use of plant nutrition which would induce expression changes of various genes.

Secondary metabolites play important roles in regulation of plant growth and defense to pests and pathogens [15]. Plants deploy numerous secondary metabolites to facilitate interaction with biotic and abiotic environment. In our study, gene expression of the three main kinds of secondary metabolites including terpenes, phenols, and alkaloids were influenced by Psa infection. Terpenes are the largest class of natural products, many of which are toxic to insects [16], fungi [17], and bacteria [18]. Expression changes of genes involved in biosynthesis of monoterpenoid, diterpenoid, triterpenoid, sesquiterpenoid, and terpenoid backbone indicated that terpenes may play an important role in interaction between kiwifruit and Psa. Changes in diterpenoid and triterpenoid were particularly important, for the former is involved in gibberellin biosynthesis, and the latter involved in brassinosteroid biosynthesis. Both of the two plant hormones were found to function in plant innate immunity [19]. Expressions of eight genes related to biosynthesis of carotenoid which is tetraterpene were all increased after Psa infection. Carotenoids are important antioxidants to sweep reactive oxygen species produced by plant under stress [20].

Plant phenols are secondary metabolites with various structures. They work as signal compounds, pigments, internal physiological regulators or chemical messengers, and function in the resistance mechanism of plants against pathogens [21]. Most phenols biogenetically arise from the shikimate-phenylpropanoid-flavonoids pathway where phenylalanine ammonia-lyase (PAL) plays the key role in phenols production. Gene expression of *PAL* was up regulated in a sample infected by Psa meanwhile the phenylpropanoid biosynthesis pathway and phenylalanine metabolism pathway were also influenced. Metabolisms of tryptophan, tyrosine, and phenylalanine which are the mean precursors of phenols were altered as well. The results above indicate that the whole metabolism pathway of phenols was regulated and plant phenols may play an important role in kiwifruit resistance to Psa.

Pathogenesis-related (PR) proteins are induced under various biotic and abiotic stresses. They play an important role in plant-defense mechanism. We identified genes encoding PR proteins belonging to 10 families of the all 17 PR protein families characterized to date [22]. Unigenes belonging to PR-1, PR-4, PR-5, PR-9, PR-10, and PR-12 showed differentially expression in Psa-infected kiwifruit. In another study on kiwifruit by Beatrice [23], PR-1 and PR-5 expression was also induced by Psa. PR-1 proteins act as a molecular marker for systemic acquired resistance response. PR-5 acts as antifungal; glucanase and xylanase inhibitors; and α-amylase and trypsin inhibitors. Its down-regulation leads to susceptibility of resist *Piper colubrinum* to the oomycete pathogen *Phytophthora capsici* [24]. PR-4 proteins bind to chitin, and play an important role in enhancing the chitinase activity. The induction of PR4 transcripts in wheat coleoptils and roots is correlated with the expression of the corresponding proteins that are expressed only in the infected tissues [25]. PR-9 catalyzes cross-linking of macromolecules in plant cell wall and produces a free radical like H_2_O_2_ against a wide range of pathogens [26]. PR-12 proteins are small cysteine rich peptides providing protection against a broad range of organisms. They are known to inhibit protein synthesis, enzyme activity and ion channel function [27]. Among these PR proteins, PR-12 protein (defensin) and PR-13 protein (thionin) also act as antimicrobial peptides which are found as host defenses against pathogens and pests in diverse organisms [28]. Genes encoding two other antimicrobial peptides hevein-like peptide and snakin peptide were found to express differentially in Psa-infected kiwifruit. Snakin peptide is involved in plant-pathogen interactions [29] and snaking-Z derived from *Zizyphus jujube* fruits displayed antimicrobial activity against different bacterial and fungal [30].

Most disease resistance genes in plants encode nucleotide-binding site leucine-rich repeat (NBS-LRR) proteins. NBS-LRR proteins are involved in detection of diverse pathogens, including bacteria, viruses, fungi, nematodes, insects, and oomycetes. Expressions of many members of the two subfamilies CNL (CC-domain-containing) and TNL (TIR-domain-containing) of NBS-LRR family were detected in kiwifruit and 70 of them were found to be regulated in the Psa-infected kiwifruit. Meanwhile, most of the differentially expressed resistance genes hold an NBS domain or an LRR domain. The NBS domain is also called NB-ARC (nucleotide binding adaptor shared by NOD-LRR proteins, APAF-1, R proteins and CED4) domain. It is thought to result in conformational changes that regulate downstream signaling [31]. The LRR domain is involved in specific recognition of pathogen effector molecules [32] and it also functions as a regulatory domain [33].

Ubiquitination regulates diverse cellular processes, including floral transition, circadian rhythm, photomorphogenesis, and cell death [34,35]. In the study of Avr9/Cf-9 interaction, Gonzálezlamothe and his colleagues [36] found that two of the three *Avr9/Cf-9 Rapidly Elicited* (*ACRE*) genes essential for *Cf*-*9*- and *Cf*-*4*-dependent hypersensitive response encode putative E3 ubiquitin ligase components. Our results identified 211 U-box domain-containing protein encoding genes, of which 25 were differentially expressed in Psa-infected kiwifruit and 22 were up regulated. U-box is a derived version of RING-finger domain that lacks the hallmark metal-chelating residues of the latter but is likely to function similarly to the RING-finger in mediating ubiquitin-conjugation of protein substrates [37,38]. *ACRE74* which encodes a U-box E3 ligase homolog was induced in Cf9 tobacco and Cf9 tomato after Avr9 elicitation and its overexpression induced a stronger HR. This shows that the E3 ligase ACRE74 is essential for plant defense and disease resistance. PUB13 (plant U-box protein 13) is a well-studied example in plant disease resistance. Silencing of the PUB13 induced spontaneous cell death, elevated resistance to biotrophic pathogens but increased susceptibility to necrotrophic pathgenes [39]. Another study showed that PUB13 is also involved in regulating the FLS2-mediated PTI [40]. In our study, most of the differentially expressed PUB encoding genes were up regulated by Psa infection in kiwifruit, indicating they may play an important role in the interaction between kiwifruit and Psa.

During the long term of interaction between plants and pathogens, plants have evolved a complete defense system, namely PTI and ETI. This immune system will be triggered by recognition of PAMPs or effector secreted by invading pathogens and induces expression of resistance genes. Meanwhile, pathogens also can escape recognition by plants by lose or change of PAMPs and disturb ETI by new evolved effectors [41]. Approximately 50 pathovars of *P. syringae* have been recognized [42], and they cause economically important diseases in a wide range of plant species. Psa was first identified in Japan in 1984 [1] and it might evolved from ancestor of other hosts [43]. Transcriptomic analysis of kiwifruit infected by Psa contributes to explore the interaction between Psa and kiwifruit. Expressions of many genes involved in PTI and ETI were detected and several important genes showed differential expression in Psa-infected kiwifruit. *CDPK* and *Rboh* were PAMP induced genes which displayed increased expression in Psa-infected kiwifruit. These two genes regulate the production of reactive oxygen species [44,45] which induce HR. WRKY TFs are a large family involved in various plant processes but most notably in coping with diverse biotic and abiotic stresses [46,47]. In this study, four WRKY genes *WRKY22*, *WRKY25*, *WRKY29*, and *WRKY33* were all up regulated by Psa infection. *WRKY22*, *WRKY29* and *WRKY33* were also found to be up-regulated in Arabidopsis induced by chitin [48]. Overexpression of *WRKY25* resulted in increased disease symptoms to *P. syringae* infection, possibly by negatively regulating salicylic acid (SA)-mediated defense responses [49]. Two *Pti* genes were induced by Psa. *Pti1* is involved in a Pto-mediated signaling pathway, probably by acting as a component downstream of Pto in a phosphorylation cascade. Its expression in tobacco plants enhanced the hypersensitive response to a *P. syringae* pv. tabacoo strain carrying the avirulence gene *avrPto* [50]. *Pti4* confers resistance to *P. syringae* pv tomato that causes bacterial speck disease in tomato [51]. RIN4 in Arabidopsis is targeted by type III effectors AvrRpt2 and AvrRpm1 which inhibit PAMP-induced signaling and compromise the host’s basal defense system. The R proteins, RPS2 and RPM1 whose encoding genes were regulated by Psa sense type III effectors-induced perturbation of RIN4 and guard the plant against pathogens [52]. One heat shock protein (HSP) encoding gene *HSP90* was also down regulated. HSP90 is required for functioning of RPS2 and its inhibition reduces the HR and abolishes resistance against *P. syringae* pv. tomato DC3000 [53].

## 4. Materials and Methods

### 4.1. Plant Materials and Treatments

The kiwifruit (*Actinidia chinensis* var. *deliciosa*) cultivar “Jinkui” kept in Institute of Botany, Jiangsu Province and Chinese Academy of Science, China, was used in this study. Shoots of good growth vigor were collected from kiwifruit trees and stuck in MS medium, and maintained in growth chambers. The condition was set with a temperature of 25 °C and 12 h/12 h (light/dark) cycles. After one week, seedlings were inoculated with the canker-causing bacteria *Pseudomonas syringae* pv. *actinidiae* (Psa). Bacterial cells were suspended in distilled water and adjusted to an OD_600_ = 0.2, and injected into the seedling stems which were carved with a knife. Five treatments were set: CK, only carved; PY3, inoculated with water and sampled 48 h after inoculation; PY4, inoculated with water and sampled at 96 h; XJ3, inoculated with Psa and sampled at 48 h; XJ4, inoculated with Psa and sampled at 96 h. Phloem of each sample was collected with three biological replicates.

### 4.2. RNA Extraction, Transcriptome Sequencing and De Novo Assembly

Total RNA was isolated from phloem samples according the method of Cai [54], and mRNA was enriched by Oligo (dT) bead. Then the enriched mRNA was fragmented into short fragments (approximately 200–700 nt) and reverse transcripted into cDNA with random primers, and then the second-strand cDNA were synthesized. Sequencing was done using Illomina HiSeq^TM^4000 by Gene Denovo Biotechnology Co. (Guangzhou, China).

After filtering of low-quality raw reads, transcriptome de novo assembly was carried out with short reads assembling program Trinity [55]. The assembled transcript whose length was larger than 200 bp was kept. The longest transcript in each locus was taken as the unigene.

### 4.3. Functional Annotation of Unigenes

BLASTx program (http://www.ncbi.nlm.nih.gov/BLAST) was used to annotate the unigenes, with an *E*-value threshold of 10^−5^ to NCBI non-redundant protein (Nr) database (http://www.ncbi.nlm.nih.gov), the Swiss-Prot protein database (http://www.expasy.ch/sprot), and the COG/KOG database (http://www.ncbi.nlm.nih.gov/COG). The best alignment results were for protein functional annotations. GO annotation of unigenes was analyzed by Blast2GO software [56], and functional classification of unigenes was performed using WEGO software [57]. Kyoto Encyclopedia of Genes and Genomes (KEGG) annotations were obtained in http://www.genome.jp/kegg.

For R-Gene analysis, protein coding sequences of unigenes were aligned by BLASTp to R-Gene database PRGdb (http://prgdb.crg.eu/wiki/Main_Page).

### 4.4. Functional Analysis of Differentially Expressed Unigenes

The unigene expression was calculated and normalized to RPKM (Reads Per kb per Million reads) [58]. The edgeR package (http://www.r-project.org/) was used to identify differentially expressed genes (DEGs) between Psa treated sample and control. Genes with a fold change ≥ 2 and a false discovery rate (FDR) ≤ 0.05 in a comparison were defined as significant DEGs. All DEGs were mapped to GO terms in the Gene Ontology database (http://www.geneontology.org), and gene numbers were calculated for every term. Significantly enriched GO terms in DEGs comparing to the genome background were defined by hypergeometric test. The rigorous FDR correction method was for *q* value correction, and GO terms were defined as being significantly enriched when the *q* value was ≤0.05. KEGG pathway enrichment analysis was done to identify significantly enriched metabolic pathways or signal transduction pathways in DEGs comparing with the whole genome background. Pathways with *q* value ≤ 0.05 were defined as significantly enriched.

### 4.5. Quantitative RT-PCR Analysis

To test the expression results from transcriptome sequencing, we determined the expression levels of 12 randomly selected unigenes through the method of qRT-PCR. Primers were designed using Primer5 software (Table S7), and *AdActin* was used as internal control. We performed qRT-PCR using the SYBR^®^ *Premix Ex Taq*^TM^ (Perfect Real Time, Dalian, China) (TaKaRa Code: DRRO41A), with PCR conditions of 40 cycles of 95 °C for 20 s, 60 °C for 20 s, and 72 °C for 40 s. Relative gene expression was calculated according to the 2^−^^△△^*^C^*^t^ method [59].

## 5. Conclusions

In order to explore the interaction between Psa and its host kiwifruit plants, we analyzed the transcriptome of kiwifruit infected by Psa. In total, 8255 differentially expressed genes were identified, including those involved in secondary metabolites metabolism, NBS-LRR protein encoding genes, and genes of plant immunity system PTI and ETI. Expression changes of genes involved in the secondary metabolism especially the biosynthesis of terpenes were evident, indicating the probable role of secondary metabolites in plant defense. Expressions of genes encoding NBS-LRR proteins which are usually products of resistance genes were also found to be regulated. Among these NBS-LRR protein genes, we noted that U-box domain containing genes were obviously differentially expressed. PUB proteins mediate ubiquitin-conjugation of protein substrates, and may function in HR. Expression of genes involved in PTI and ETI was detected and some key genes showed differential expression in Psa-infected kiwifruit. These genes play important roles in plant immunity system, such as PAMP and effector recognition, signal transduction, HR and defense related gene induction. We hope our results will facilitate the future study of interaction between Psa and kiwifruit.

## Figures and Tables

**Table 1:** Functional annotation of the kiwifruit unigenes.

Database	Number of Unigenes	Percentage
Nr	49,897	45.31
Swissprot	34,331	31.17
KOG	30,430	27.63
KEGG	20,524	18.64
Annotation gene	50,305	45.68
Without annotation gene	59,829	54.32
Total unigenes	110,134	100.00

Nr: non-redundant protein sequence; KOG: Clusters of eukaryotic ortholog groups of proteins; KEGG: Kyoto encyclopedia of genes and genomes.

**Table 2:** The enriched differential progress top 20.

GO ID	Description	DEGs Genes with Pathway Annotation (1521)	All Genes with Pathway Annotation (24,936)
1	Terpene biosynthetic process	7	7
2	Tricarboxylic acid metabolic process	20	120
3	Regulation of defense response	15	77
4	Terpene metabolic process	7	19
5	Citrate metabolic process	19	119
6	Regulation of response to stress	15	84
7	Acetate metabolic process	5	18
8	Positive regulation of microtubule Polymerization or depolymerization	2	2
9	Metabolic process	1203	19,066
10	Lignin metabolic process	5	21
11	Apoptotic process	2	3
12	Regulation of cell size	2	3
13	phenylpropanoid metabolic process	15	128
14	Cellular amino acid catabolic process	6	33
15	Response to bacterium	27	282
16	Energy coupled proton transmembrane Transport, against electrochemical gradient	11	88
17	Nucleoside diphosphate metabolic process	5	26
18	Cellular respiration	11	89
19	Regulation of multi-organism process	3	10
20	Arachidonic acid metabolic process	2	4

**Table 3:** Enriched pathways involved in secondary metabolism.

Metabolites	Pathway ID	Pathway	DEGs Genes with Pathway Annotation (925)	All Genes with Pathway Annotation (11,433)
Terpenes	ko00902	Monoterpenoid biosynthesis	8 (0.86%)	17 (0.15%)
	ko00903	Limonene and pinene degradation	8 (0.86%)	32 (0.28%)
	ko00904	Diterpenoid biosynthesis	8 (0.86%)	32 (0.28%)
	ko00909	Sesquiterpenoid and triterpenoid biosynthesis	4 (0.43%)	17 (0.15%)
	ko00906	Carotenoid biosynthesis	8 (0.86%)	51 (0.45%)
	ko00905	Brassinosteroid biosynthesis	2 (0.22%)	17 (0.15%)
	ko00900	Terpenoid backbone biosynthesis	9 (0.97%)	117 (1.02%)
Phenols	ko00940	Phenylpropanoid biosynthesis	64 (6.92%)	317 (2.77%)
	ko00380	Tryptophan metabolism	28 (3.03%)	105 (0.92%)
	ko00350	Tyrosine metabolism	14 (1.51%)	86 (0.75%)
	ko00360	Phenylalanine metabolism	9 (0.97%)	84 (0.73%)
	ko00941	Flavonoid biosynthesis	9 (0.97%)	91 (0.8%)
	ko00944	Flavone and flavonol biosynthesis	1 (0.11%)	11 (0.1%)
	ko00400	Phenylalanine, tyrosine and tryptophan biosynthesis	6 (0.65%)	89 (0.78%)
Alkaloids	ko00950	Isoquinoline alkaloid biosynthesis	7 (0.76%)	39 (0.34%)
	ko00960	Tropane, piperidine and pyridine alkaloid biosynthesis	4 (0.43%)	42 (0.37%)

**Table 4:** Differentially expressed genes encoding pathogenesis-related proteins.

Family	Properties	All Expressed Unigenes	Differentially Expressed Unigenes
PR-1	Unknown	6	2
PR-2	β-1,3-glucanase	1	0
PR-4	Chitinase type I, II	2	1
PR-5	Thaumatin-like	39	6
PR-6	Proteinase-inhibitor	9	0
PR-9	Peroxidase	96	8
PR-10	“Ribonuclease-like”	1	1
PR-12	Defensin	3	1
PR-13	Thionin	3	0
PR-14	Lipid-transfer protein	21	0

**Table 5:** The differentially expressed resistant genes.

Class	Number of All Identified Resistant Gene	Number of Differentially Expressed Genes
CN	123	6
CNL	411	25
L	19	0
Mlo-like	34	0
N	706	36
NL	828	69
Other	93	8
PTO	2	1
Pro-like	74	4
RLK	281	20
RLK-GNK2	246	13
RLK-Kinase	1	0
RLK-Malectina	1	0
RLK-Pro-like	1	0
RLP	1265	60
RLP-Malectin	5	0
RLP-Malectina	1	0
RPW8-NL	11	1
T	98	5
TNL	572	35
TNL-OT	1	0

## Supplementary Materials

Supplementary materials can be found at http://www.mdpi.com/1422-0067/19/2/373/s1.
