# Supplementary material for: Transcriptome Analysis of Kiwifruit in Response to Pseudomonas syringae pv. actinidiae Infection
Source: Int J Mol Sci. 2018 Jan 26;19(2):373. doi: 10.3390/ijms19020373 (PMC5855595; doi:10.3390/ijms19020373)
Supplement: Supplementary file 1 [file ijms-19-00373-s001.zip › Supplementary final done ijms-249570/Figure S1, S2.pdf]

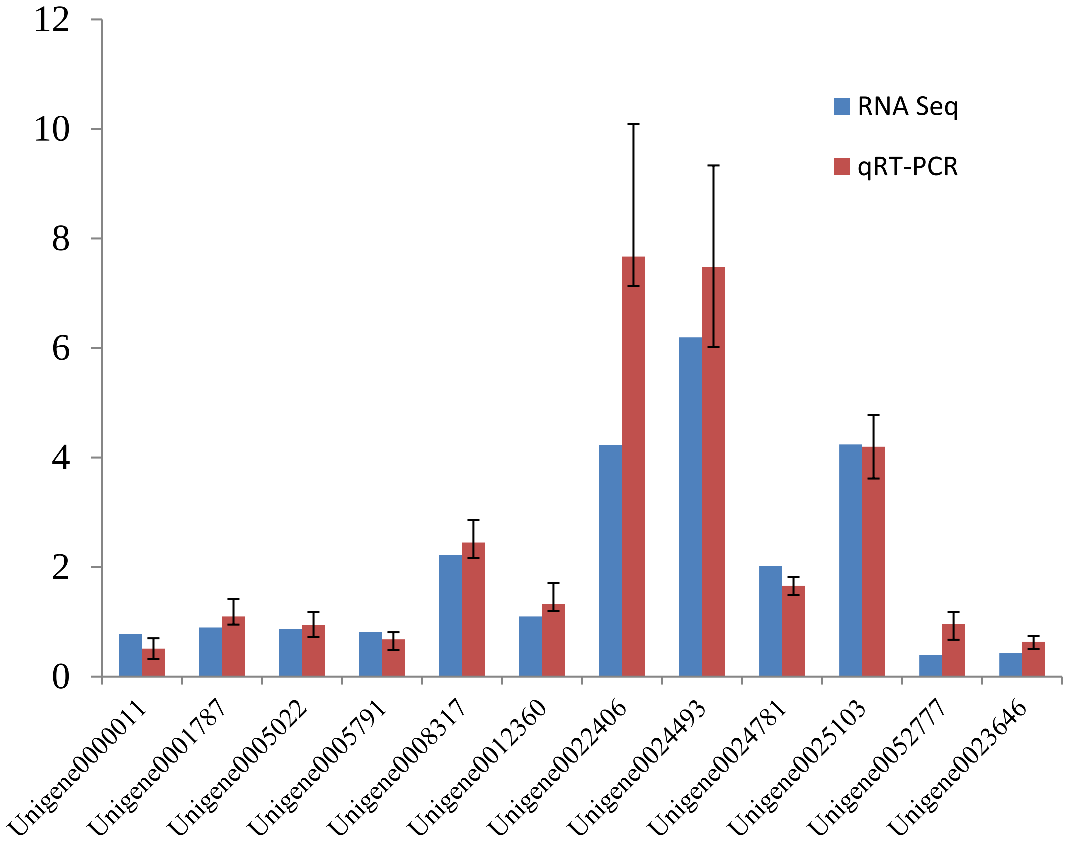

Figure S1. Expression levels of the selected unigenes from RNA sequencing data and qRT-PCR. Expression values were displayed by the fold of XJ3 to PY3.

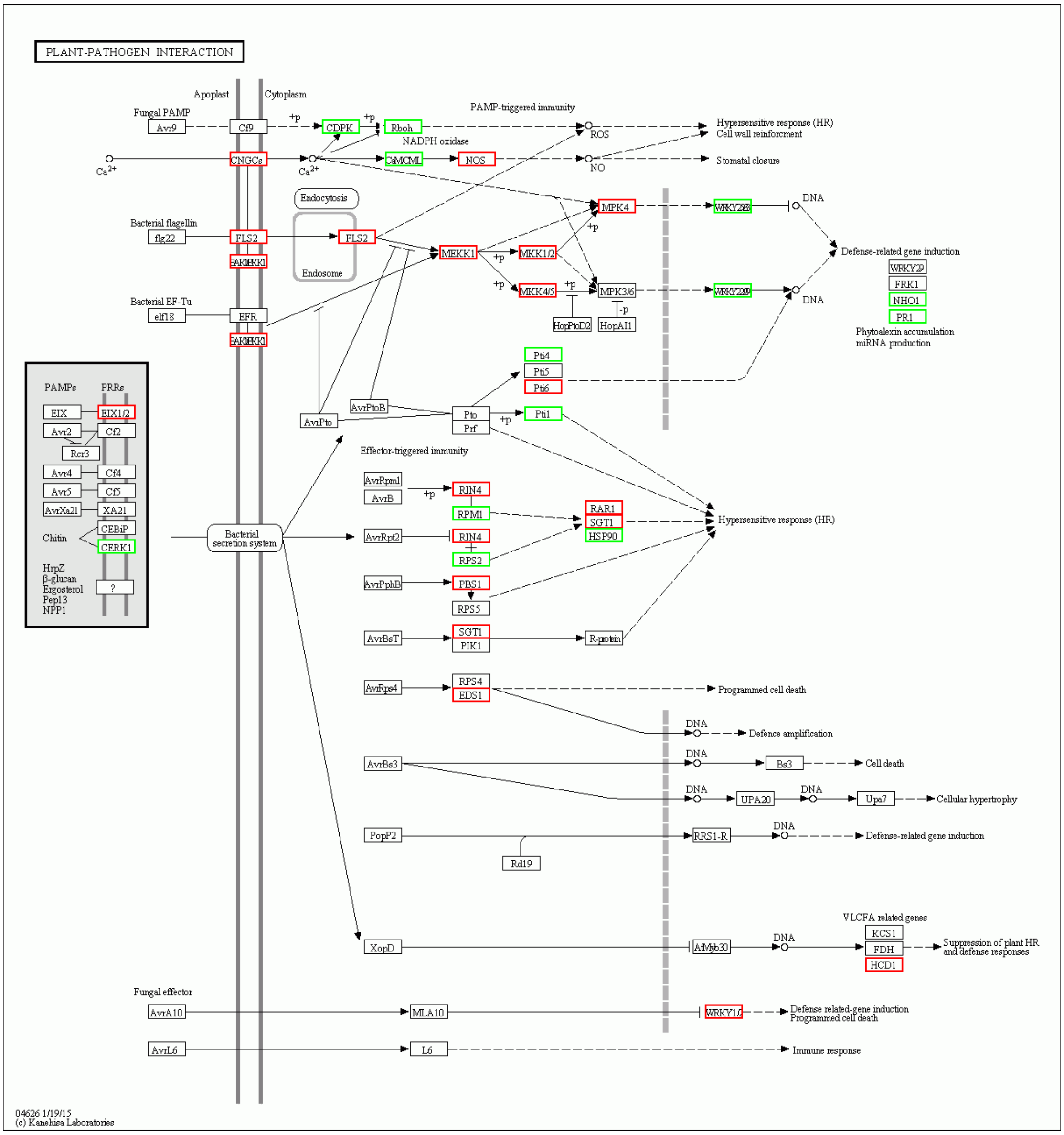

Figure S2. Expression of genes in the plant-pathogen interaction pathway. Genes in green frame were expressed differentially in Psa infected kiwifruit and genes in red frame were not.
